# Influence of Mediterranean Diet on Sexual Function in People with Metabolic Syndrome: A Narrative Review

**DOI:** 10.3390/nu16193397

**Published:** 2024-10-06

**Authors:** Vittorio Oteri, Francesco Galeano, Stefania Panebianco, Tommaso Piticchio, Rosario Le Moli, Lucia Frittitta, Veronica Vella, Roberto Baratta, Damiano Gullo, Francesco Frasca, Andrea Tumminia

**Affiliations:** 1 Endocrinology Section, Department of Clinical and Experimental Medicine, Garibaldi-Nesima Hospital, University of Catania, 95122 Catania, CT, Italy; research@droteri.it (V.O.); francesco.galeano3@gmail.com (F.G.); dottoressa.panebianco@gmail.com (S.P.); tommaso.piticchio@unikore.it (T.P.); rosario.lemoli@unikore.it (R.L.M.); lucia.frittitta@unict.it (L.F.); veronica.vella@unict.it (V.V.); 2 Department of Medicine and Surgery, University Kore of Enna, 94100 Enna, EN, Italy; 3 Diabetes and Obesity Center, Garibaldi-Nesima Hospital, University of Catania, 95122 Catania, CT, Italy; 4 Endocrine Unit, Garibaldi-Nesima Hospital, 95122 Catania, CT, Italy; rob.baratta@gmail.com (R.B.); gullo.family@alice.it (D.G.); andreatumminia82@gmail.com (A.T.)

**Keywords:** metabolic syndrome, sexual function, Mediterranean diet, erectile dysfunction, libido, infertility, menstrual cycle, oxidative stress, obesity, metabolism

## Abstract

Metabolic syndrome (MS), a cluster of cardiometabolic disorders, and sexual dysfunction are two conditions that impact a large proportion of the general population. Although they can occur independently, they are frequently linked and significantly affect people’s quality of life. In recent years, research has increasingly focused on the importance of diet, particularly the Mediterranean diet (MD), in modulating sexual function due to its anti-inflammatory, antioxidant, and vasodilatory properties. In this narrative review, we examined the relationship between MS and sexual function in both men and women, with a special emphasis on the MD’s therapeutic efficacy in improving sexual dysfunction. In men, MD has been shown to ameliorate erectile dysfunction, as well as several sperm parameters, perhaps leading to improved fertility. On the other hand, adherence to MD has been demonstrated to partially recover several sexual dysfunctions in women, such as those related to their menstrual cycle, menopause, endometriosis, and polycystic ovary syndrome. These favorable effects of MD have been demonstrated in both sexes also among people affected by MS. However, more targeted studies are needed to validate these data for different dietary approaches as well.

## 1. Introduction

Metabolic syndrome (MS) is defined as a combination of several cardiometabolic conditions, including hyperglycemia, abdominal obesity, insulin resistance (IR), hypertension, hypertriglyceridemia, and low levels of high-density lipoprotein (HDL) [1].

It is estimated that MS affects nearly one-third of the world’s population, with projections suggesting that the prevalence will rise to more than half of the global population in the next 20 years [1,2]. A meta-analysis of global data from 28 million individuals revealed notable variations in the worldwide prevalence of MS on geographic region, age, and income level of countries. The highest prevalence of MS was found in the Eastern Mediterranean Region (36.6%), while the lowest was observed in the Africa Region (23.1%). Among younger populations, the prevalence of MS was approximately 3% in children and 5% in adolescents, with some differences across countries [3,4]. These alarming data claim effective interventions to reduce the global burden of MS and its associated conditions. However, available data show considerable variability in the prevalence of MS depending on the diagnostic criteria used. For instance, in a study involving 246 overweight or obese children and adolescents, 37.4% of them were classified as metabolically healthy based on the absence of clinical MS criteria; however, while considering the presence of insulin resistance (HOMA-IR < 2.5) in addition to the canonical MS criteria, this percentage dropped to 10.6%. This phenomenon remarks the crucial role of insulin resistance as one of the main etiological features leading to metabolic impairment in MS individuals, irrespective of the excess adipose tissue [5].

Among others, sexual dysfunctions should be mentioned as one of the most impactful negative effects of MS on human health status [6]. Male and female sexual dysfunctions are widespread, affecting 52% of men over 40 years of age and up to 75% of women aged 40–50 years [6,7]. Although MS and sexual dysfunction can occur independently, they are frequently interconnected [8], and both significantly influence individuals’ quality of life. As the global incidence of MS increases, many more individuals may experience various degrees and types of sexual impairment in future years.

Lifestyle modification, including healthy nutritional therapy and structured physical exercise, should be the first-line treatment strategy for MS and its related clinical consequences [9].

Evidence from both observational studies and clinical trials generally supports the positive impact of the Mediterranean diet (MD) both on MS [10,11] and on cardiovascular health, which are crucial for individuals’ sexual function. As shown by the ATTICA study, a 10% increase in adherence to the MD is linked to a 15% reduction in the odds of developing cardiovascular disease (CVD). Furthermore, the presence of MS doubles the odds of CVD for those with low adherence to the diet [12].

Cardiovascular performance is a critical factor in sexual function, as CVD and its risk factors are closely linked to the development of sexual dysfunction [13].

The MD is characterized by its emphasis on plant-based foods, such as vegetables, fruits, nuts, whole grains, and legumes, while also incorporating moderate amounts of dairy, poultry, eggs, and seafood. It prioritizes unsaturated fats, mainly from olive oil and nuts, replacing saturated and trans fats known to contribute to CVD. The benefits of the MD extend beyond its macronutrient composition to specific bioactive compounds, including dietary flavonoids, phenols, sterols, omega-3 fatty acids (n-3 FAs), and fiber. These components are particularly important in reducing oxidative stress and inflammation, two key contributors to the pathophysiology of MS and CVD [14].

C-reactive protein (CRP) and interleukin-6 (IL-6) have been identified as strong predictors of both diabetes and CVD in prospective cohort studies [11]. CRP levels are influenced by inflammatory cytokines such as IL-6, which may play a role in the development of atherosclerosis by activating immune cells and causing endothelial dysfunction [10]. Studies show that individuals with higher adherence to the MD tend to have lower levels of CRP, IL-6, and fibrinogen, which are important markers of inflammation [11].

One of the key components of the MD is olive oil (OO), particularly extra virgin olive oil (EVOO), which is rich in monounsaturated fatty acids (MUFAs), polyunsaturated fatty acids (PUFAs), tocopherols, and bioactive compounds like phenols and sterols. The most abundant polyphenols in olive oil, such as oleuropein, hydroxytyrosol, and tyrosol, have been shown to possess cardioprotective properties due to their antioxidant and anti-inflammatory effects. Regular consumption of EVOO has been associated with a significant reduction in cardiovascular events, including an 82% relative reduction in the risk of a first myocardial infarction (MI), a 14% lower risk of CVD, and an 18% lower risk of coronary heart disease [15]. Moreover, EVOO has anti-hypertensive effects by stimulating nitric oxide (NO) production and inhibiting the expression of endothelin-1, which helps lower blood pressure and slow the progression of atherosclerosis [15].

Other Mediterranean oils, such as those from nuts and seeds, are also rich in tocopherols, phytosterols, and polyphenols, contributing to reduced oxidative stress and maintaining the functionality of organelles such as mitochondria. Additionally, phytosterols found in Mediterranean oils may play a role in preventing or treating age-related diseases and CVD [16].

EVOO can also modify the lipid composition of very low-density lipoproteins (VLDLs), particularly by modulating the incorporation of lipids into VLDL particles, thus reducing triglyceride levels [17].

Walnuts, another staple of the MD, are rich in L-arginine, a precursor of nitric oxide, and alpha-linolenic acid, a plant-based omega-3 fatty acid. Both compounds contribute to the anti-hypertensive and anti-atherogenic properties of the MD. The high fiber content of the diet, along with omega-3 fatty acids and antioxidants from foods like olive oil, legumes, whole grains, fruits, and vegetables, also plays a crucial role in reducing the risk factors associated with MS [10].

Furthermore, adjusting the fiber content in meals can significantly affect inflammatory markers, helping to mitigate the temporary oxidative stress that arises after consuming macronutrients [14,18].

Given the close relationship between MS and sexual dysfunction, our work aims to review, in a narrative fashion, the interplay between MS and sexual dysfunctions both in men and in women, with a particular focus on the therapeutic efficacy of MD in improving sexual function. The comprehensive benefits of the MD, from its bioactive compounds to its balanced macronutrient profile, support its role in mitigating the effects of MS, improving cardiovascular health and ultimately enhancing sexual well-being.

## 2. Methods

We searched the MEDLINE database via PubMed up to the 25th of September 2024. To achieve the maximum sensitivity of the search strategy, we combined the terms referring to the following major topics: metabolic syndrome, Mediterranean diet, dietary approaches, sexual function, and dysfunction. We tailored searches using appropriate controlled vocabulary indexing and natural language search terms; the full search strategy for each section of the review is available in Appendix S1 of Supplementary Materials. The reference lists of the included articles and previous literature reviews on the topic were reviewed for further identification of potentially relevant studies to ensure the completeness of this review.

Eligible studies for our narrative review included those investigating the relationships between the Mediterranean diet or other dietary approaches, sexual function and dysfunction, and metabolic syndrome. We included the most relevant experimental, observational, and review studies published in peer-reviewed journals and written in English. We also excluded studies in which data were not accessible, missing, without an available full text, or not well reported.

All data were extracted from articles’ text, tables, and figures using the Population, Intervention, Comparison, and Outcome (PICO) framework [19] and included at least the following: title, authors, year of publication, study design, study population, outcomes, and main results.

## 3. Interconnections between Sexual Function and Metabolic Syndrome

Sexual function is characterized by different phases: desire, arousal, and orgasm. Sexual dysfunction is a disorder that affects sexual pleasure, orgasm, or sexual desire, or causes pain during sexual intercourse. It represents a medical and psychological condition that can affect self-esteem, body image, interpersonal relationships, and physical health in general, including fertility.

In men, sexual dysfunction is more prevalent among homosexuals (42–79%) and those with infertility (12%) [20]. These dysfunctions can manifest as erectile dysfunction (ED), sexual anxiety, pain during intercourse or anodyspareunia, lack of pleasure during sex, premature ejaculation (PE), hypoactive sexual desire, anorgasmia, and other orgasmic difficulties [20]. In infertile men, the most common types of sexual dysfunction are hypoactive sexual desire and lack of sexual satisfaction, with prevalence rates ranging from 8.9% to 68.7% [21]. ED and/or PE, assessed using validated tools, affect one in six infertile men, while orgasmic dysfunction is found in one in ten [21].

Female sexual dysfunction (FSD) seems to affect about 40% of women [22], although this estimate might be underestimated due to underreporting. The Female Sexual Function Index (FSFI) is commonly used in these cases, assessing six domains: desire, arousal, lubrication, orgasm, satisfaction, and pain [22]. Other conditions related to FSD include premenstrual syndrome (PMS), dysmenorrhea, endometriosis (EM), polycystic ovary syndrome (PCOS), and infertility. Table 1 summarizes the main classifications of sexual dysfunctions and other relevant medical conditions according to the guidelines of the International Classification of Diseases, 10th Edition (ICD-10), and the Diagnostic and Statistical Manual of Mental Disorders, 5th Edition (DSM-5) [23,24].

### 3.1. Points of Variability in the Prevalence Rate of Metabolic Syndrome and Sexual Dysfunction

The prevalence of metabolic syndrome and sexual dysfunction exhibits significant variability across different populations, largely influenced by a combination of personal, demographic, and social factors. Understanding these variables is essential to interpreting the results of studies, as the heterogeneity of populations complicates the reproducibility of findings.

Personal factors, such as age, medical history, and individual metabolic status, are among the most important predictors. Age plays a crucial role; metabolic syndrome is significantly more prevalent among older adults, as the risk for conditions like hypertension, insulin resistance, and abdominal obesity increases with age [25]. Similarly, sexual dysfunction, including erectile dysfunction (ED) in men and hypoactive sexual desire disorder in women, becomes more common with advancing age, driven by both physiological changes and chronic disease prevalence [26,27]. Medical history is another critical aspect, as individuals with conditions such as type 2 diabetes, cardiovascular disease, and obesity—common components of metabolic syndrome—are at a significantly higher risk of developing sexual dysfunction. For instance, studies show that men with diabetes are two-to-three times more likely to develop erectile dysfunction compared to non-diabetic men [28], while women with metabolic syndrome may experience reduced libido and sexual satisfaction due to hormonal imbalances and vascular insufficiencies [29].

In terms of demographic factors, geographic variability plays a notable role in the prevalence of both metabolic syndrome and sexual dysfunction. The incidence of metabolic syndrome can vary widely between regions due to differences in dietary habits, levels of physical activity, and healthcare systems. For example, populations in Western nations tend to exhibit higher rates of metabolic syndrome, largely due to the prevalence of high-calorie, processed foods and sedentary lifestyles [30]. Conversely, in developing countries, urbanization is associated with rising rates of metabolic syndrome, as traditional diets are replaced with Westernized dietary patterns [31]. Geographic factors also influence the prevalence of sexual dysfunction, as cultural attitudes toward sexual health, stigmatization of sexual issues, and access to medical interventions such as phosphodiesterase inhibitors (for erectile dysfunction) differ across countries [32]. In regions with less access to healthcare, conditions like ED may be underdiagnosed and undertreated, further skewing prevalence rates [33].

Social factors, such as socioeconomic status, income, and education level, have a profound impact on both metabolic health and sexual function. Individuals with lower incomes often face barriers to maintaining a healthy lifestyle, including limited access to nutritious food and healthcare, thus contributing to higher rates of obesity, diabetes, and metabolic syndrome [34]. For example, individuals in lower socioeconomic brackets may have reduced access to healthcare resources like counselling, hormone replacement therapies, or medications for erectile dysfunction, exacerbating the prevalence of sexual dysfunction in these groups [35]. Education also plays a role, as lower education levels are often associated with poorer health literacy, leading to lower rates of disease prevention and management of metabolic syndrome and sexual dysfunction [36].

The heterogeneity of populations in terms of age, health status, geographic location, and socioeconomic factors significantly affects the generalizability and reproducibility of research findings. Studies that focus on specific subpopulations may yield results that are not directly applicable to other groups, given the wide variation in lifestyle, healthcare access, and social determinants of health. For example, findings from a study conducted in a high-income urban setting may not translate well to a low-income rural population, where access to healthcare and lifestyle factors are substantially different. As a result, future research should aim to account for this heterogeneity, ensuring that a diverse range of personal, demographic, and social variables are considered to improve the reliability and applicability of the findings.

### 3.2. Male Sexual Function and Metabolic Syndrome

ED is supposed to reach a worldwide prevalence of 322 million cases by 2025 [37]. Approximately 80% of men experiencing ED are either overweight or obese, with obese men facing a 30% greater risk of sexual dysfunction compared to those with a normal body mass index [38]. Excess body weight leads to increased fat stores, resulting in decreased levels of both free and total testosterone [39]. Lower levels of testosterone are also associated with reduced libido [39], and meta-analyses show that testosterone supplementation can improve libido and erectile function in obese men with low-to-low–normal testosterone levels [40]. Visceral adipose tissue (VAT) should be considered an endocrine organ that can influence gonadal steroidal hormones through the enzyme aromatase, which plays a key role in converting androgens to estrogens and may lead to secondary hypogonadism by suppressing the reproductive axis, reducing total testosterone (TT), and sex hormone-binding globulin (SHBG) serum concentrations [41,42]. Adipocytes can also influence TT serum levels via leptin production interacting with a leptin receptor isoform expressed on Leydig cells [43]. In men (especially in obese individuals), nitric oxide (NO) levels are reduced, leading to impaired vasodilatation and penile erection [44]. Elevated concentrations of free radicals also contribute to atherosclerotic damage and the critical stenosis of cavernous arteries [45]. Hyperglycemia, another critical aspect of MS, triggers a cascade of cellular events that increase the production of reactive oxygen species and oxygen-derived free radicals, contributing to increased oxidative stress. Additionally, hyperglycemia can lead to the glycation of penile cavernosal tissue, compromising collagen production and potentially contributing to ED [46]. MS, along with obesity, overweight, and hyperglycemia, is characterized by a proinflammatory state that can lead to ED. In these patients, increased levels of cytokines, like IL-1β, IL-6, and TNF-α, inhibit testosterone production by interfering with steroidogenesis in Leydig cells [43].

It has been reported [28] that nearly 40% of men with ED meet the US National Cholesterol Education Program criteria for MS [47]. Another study found that, among 236 patients with MS, 96.5% reported ED, 39.6% had hypoactive sexual desire, 22.7% had premature ejaculation, and 4.8% had delayed ejaculation. Additionally, hypogonadism was observed in 11.9% of patients with MS compared to only 3.8% of the remaining participants [48].

Recently, another aspect that has been evaluated is the influence of MS on male fertility. Various mechanisms can be implicated: altered hormone levels, increased scrotal temperature due to increased gonadal fat, pro-inflammatory state, and oxidative stress [49]. However, the link between male infertility and MS remains controversial: some studies did not show any significant relationship between MS and sperm parameters [50,51], even among men attending fertility centers [52,53,54]. Other studies, however, found a negative association between MS and various sperm parameters, including sperm count, concentration, motility, and morphology [54,55,56,57,58,59,60]. Inconsistencies in the results of these studies could be explained through the small sample sizes and their monocentric design. More specific studies are needed to define the exact role of MS on male reproductive health.

### 3.3. Female Sexual Function and Metabolic Syndrome

Women with MS are more likely to experience sexual dysfunction than those without. It has been reported that women with MS had significantly lower scores for arousal, orgasm, and lubrication compared to controls [61].

The pro-inflammatory environment associated with MS is also involved in sexual disorders in women. Esposito et al. demonstrated that the Female Sexual Function Index (FSFI) score was negatively correlated with CRP levels [62].

The negative link between MS and fertility has been evaluated by a randomized controlled trial conducted among 1508 infertile Chinese women with PCOS. The study demonstrated that those participants with MS had a longer history of infertility, a greater number of unsuccessful in vitro fertilization (IVF) cycles, and fewer retrieved oocytes and embryos for transfer. Cumulative live birth rates were also impaired in women with MS. Every feature of MS has a detrimental effect on female fertility probably through chronic inflammation and endothelial damage, which hinder oocyte development and endometrial receptivity [63].

EM is associated with metabolic diseases such as diabetes [64], hypercholesterolemia [65], and elevated triglycerides levels [66], as well as hypertension [65] and elevated waist circumference [67]; Li B. et al. found also other links between EM and features of MS [68], likely due to the consistent inflammatory response in this disease. They found that MS is more common in women who undergo surgery, hysterectomy, or oophorectomy.

Another condition often associated with endometriosis is dyspareunia, which is characterized by ongoing or recurring pain during sexual activity that can cause significant distress [69]. This often increases the likelihood of developing sexual dysfunction, relationship difficulties, a lower quality of life, anxiety, and depression. Aside from endometriosis, common triggers for dyspareunia include vulvodynia, insufficient lubrification, vaginal atrophy, postpartum changes, pelvic floor disorders, and vaginismus [69,70]. In some cases, no obvious cause is apparent, and emotional factors might be important [24]. It is also a common issue in postmenopausal women [70].

Postmenopausal women have a risk over 60% of developing MS [71], with a slightly higher prevalence in older women compared to men, probably due to menopause, loss of ovarian function, hormonal changes, and increase in BMI, especially in cases of surgical menopause [72]. Estrogens play an important metabolic role, interfering with glucose metabolism, and their decline during menopause can lead to insulin resistance [73]. In addition, menopause can increase abdominal fat, inflammatory state, and blood pressure [74]. Other hormonal changes involved in menopause, such as the decrease in adiponectin levels, may also influence changes in glucose and lipid metabolism [75]. Postmenopausal women have a higher prevalence of impaired glucose tolerance (IGT) [76,77], hypertriglyceridemia [77,78], and hypertension [72] compared to premenopausal women, along with increased waist circumference and BMI [72]. Women who undergo natural or surgical menopause and use postmenopausal hormone therapy have a lower risk of developing MS compared to those who do not [77,79].

PCOS is a common endocrine disorder with a prevalence of approximately 6–20% among women of reproductive age [80]. It exhibits great heterogeneity in clinical expression and metabolic disorders [81], such as insulin resistance, increased waist circumference, and dyslipidemia, which can lead to MS. The prevalence of MS is reported to be 43% among adult women and 30% among adolescents with PCOS. Insulin resistance, a common issue in PCOS patients, affects 50–80% of this subgroup [82], and it causes compensatory hyperinsulinism and low insulin sensitivity in peripheral tissues such as skeletal muscle and adipose tissue [83]. Increased BMI and visceral adipose fat are commonly found in women with PCOS, along with adipose tissue endocrine dysfunction and a pro-inflammatory role, which may contribute to worsening insulin resistance [84] and ovarian function [85]. The prevalence of dyslipidemia in PCOS is 70%, with higher serum levels of low-density lipoprotein cholesterol (LDL) and triglycerides and lower levels of HDL-cholesterol [86]. Women with PCOS may suffer from cardiovascular diseases such as hypertension, atherosclerosis, and coronary heart disease as a consequence of insulin resistance, obesity, dyslipidemia, and androgen excess [87].

**Figure 1:**
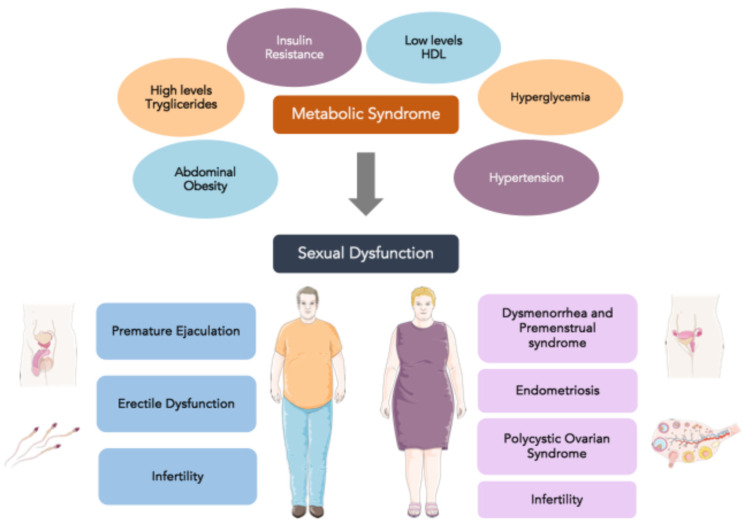
Interconnections between metabolic syndrome and sexual dysfunctions in men and women.

Figure 1 depicts the interconnections between MS and sexual dysfunctions in men and women.

## 4. Impact of the Mediterranean Diet on Metabolic Syndrome

The MD is a nutritional approach inspired by the traditional eating patterns of countries bordering the Mediterranean Sea, such as Greece, Southern Italy, and Spain. It is renowned for its numerous health benefits, particularly in promoting cardiovascular health and managing metabolic conditions [88].

MD is founded on plant-based foods: vegetables, fruits, herbs, nuts, beans, and whole grains. Moderate amounts of dairy, poultry, and eggs are part of the MD, as is seafood. In contrast, red meat is consumed only occasionally. Unsaturated fats are a key component of the MD, replacing saturated and trans fats, which can contribute to CVD. Olive oil and nuts are the main sources of fat in the MD, providing unsaturated fat. When unsaturated fat comes from plant sources, it appears to lower levels of total cholesterol, as well as LDL [89]. Protein sources in the MD include lean options like fish and seafood, which are eaten at least twice a week and are rich in n-3 FAs. Poultry is consumed in moderation, while legumes like beans, lentils, and chickpeas serve as important plant-based protein sources. Dairy products, mainly cheese and yoghurt, often derived from sheep or goats, are consumed with moderation. Red meat is limited in this diet. Eggs must be eaten with moderation, typically a few times per week. Red wine could also be considered a part of the MD if consumed with moderation and usually with meals [90].

Calories in MD are provided approximately 45% by carbohydrates; 15% by proteins; and 35–45% by fats, of which about 20% derives from monounsaturated fatty acids (MUFAs), 5% from polyunsaturated fatty acids (PUFAs), and 9% from saturated fatty acids (SFAs) [91].

The various dietary nutrients and bioactive substances of the MD have shown positive effects on the different components of MS [88,92]. Over the years, a substantial number of studies have been conducted on this topic. A systematic review and meta-analysis by Bakaloudi et al. [93] analyzed the impact of different degrees of adherence to MD on individuals’ MS parameters. All the studies reviewed showed a lower percentage of individuals with a waist circumference >102 cm for males and >88 cm for females in groups with higher adherence to MD [94,95]. Significant positive outcomes were found for HDL cholesterol concentration (and overall lipid profile), confirming previously reported data from other randomized controlled trials [94,96,97]. This phenomenon may be due to reduced visceral fat and a higher intake of olive oil and polyphenols, antioxidants, and MUFA that influence the composition of HDL cholesterol particles and fat metabolism [98]. Additionally, oils rich in n-3 FAs, particularly those found in fish, cause a significant decrease in triglycerides by reducing postprandial elevation, enhancing clearance, and suppressing the activity and synthesis of lipoprotein lipase [99]. An inverse but non-statistically significant association was found between elevated blood pressure and adherence to the MD; the health benefits were evident in both systolic and diastolic blood pressure levels [100]. On the other hand, a meta-analysis conducted on fasting glucose alterations showed no significant benefits in patients with high adherence to the MD. A possible explanation for this outcome could be the high number of individuals diagnosed with diabetes or at diabetic risk who participated in the included studies [101].

## 5. Impact of the Mediterranean Diet on Sexual Function

The Mediterranean Diet and Type 2 Diabetes (MÈDITA) trial [102] was the first randomized clinical trial to assess the autonomous impact of the MD on sexual health [8] compared to a low-fat diet. The trial involved 215 individuals who had recently been diagnosed with type 2 diabetes (T2D). The results indicated that participants in the MD group experienced significantly smaller declines in erectile function (IIEF) for men and FSFI for women, compared to those in the low-fat diet group. To be noted, the trial was unblinded, and the study did not initially plan to evaluate sexual function, which might have led to imbalances between the groups that were not accounted for in the original design.

Along with these findings, a study on 595 sexually active women with T2D demonstrated an inversely proportional relationship between adherence to the MD and the prevalence of sexual dysfunctions. These results suggest a potential protective effect of the MD against sexual dysfunction in individuals with T2D [103].

### 5.1. Male Sexual Dysfunction

#### 5.1.1. Erectile Dysfunction

ED is defined by the Fourth International Consultation on Sexual Medicine as the consistent or recurrent inability to achieve and/or maintain a penile erection sufficient for sexual satisfaction [24].

A comprehensive review highlighted that nutritional approaches rich in plant foods like the MD are abundant in polyphenols, which can enhance NO availability. Additionally, omega-3 fatty acids from fish contribute to higher NO levels, further supporting the beneficial effects of the MD on erectile function. Conversely, high-fat diets are associated with a higher incidence of ED [104,105]. Various studies have examined the link between different types of diets and ED. For example, a prospective study involving 21,469 men found that strong adherence to the MD was inversely associated with ED [106]. Similarly, a case–control study comparing 100 men with ED to 100 age-matched controls without ED found a significant inverse relationship between MD adherence and ED [107]. Since diabetes is also a significant risk factor for the development of ED, several studies have investigated the correlation between dietary approaches in patients with diabetes and ED: for example, a study on 555 men with T2D found that those with the highest adherence to the MD had a lower prevalence of overall ED and severe ED compared to those with low adherence [108].

Moreover, the FERTINUTS study [109] examined the impact of nut supplementation on erectile and sexual function in 83 healthy young men, observing that those who consumed 60 g of mixed nuts daily showed significant improvements in orgasmic function and sexual desire; however, no significant changes were observed in erectile function compared to the control group, who followed a Western-style diet without nuts [110].

#### 5.1.2. Male Infertility

Male infertility is frequently characterized by three key conditions: oligospermia (low sperm concentration), asthenozoospermia (reduced or absent sperm motility), and teratozoospermia (abnormal sperm morphology). These issues collectively account for over 90% of male infertility cases. Recent research underscores the significant role of nutrition in male reproductive health, revealing a clear connection between dietary habits and fertility outcomes [111].

Adherence to the MD has been linked to better semen parameters, including higher sperm concentration and motility [112]. Also, dietary fatty acids, especially omega-3 polyunsaturated fatty acids, are vital for maintaining healthy sperm profiles and enhancing fertility. While omega-3s generally improve sperm quality, saturated and trans fats have detrimental effects [113].

The Western diet, rich in processed foods and saturated fats, is associated with poorer sperm quality and reduced fertility [114]. However, some studies indicate that adding walnuts to a Western diet can improve sperm vitality, motility, and morphology [115]. Vegetarian and vegan diets show mixed results regarding fertility. They may lack essential nutrients, like iron and certain fatty acids, which can potentially affect fertility [114,116,117]. In addition, Orzylowska et al. evaluated the sperm characteristics of 26 vegetarians, 5 vegans, and 443 non-vegetarian males, and they found that vegetarians and vegans had lower sperm concentrations and lower total motility compared to non-vegetarians [118]. Moreover, a meta-analysis found no significant differences in semen quality parameters, including total sperm count, progressive and total motility, sperm morphology, and concentration, between vegetarians and omnivores [119]. Another study compared sperm quality parameters in vegans and non-vegans, showing that the former had a higher total sperm count, a better percentage of rapid progressively motile sperm, and a lower proportion of spermatozoon with DNA damage [120]. These mixed findings highlight the need for further research to fully understand the relationship between plant-based diets and fertility.

### 5.2. Female Sexual Dysfunction

#### 5.2.1. Menstrual Cycle

To date, there is only one longitudinal cohort study that has investigated the association between adherence to the MD and the age of menarche [121]. The study suggested that girls with higher adherence to the MD had a 55% lower risk of experiencing early menarche compared to those with lower adherence. The potential mechanism by which the MD may influence the timing of menarche could be related to the level of SHBG and endogenous estrogens in women [22].

The menstrual cycle is a critical indicator of reproductive health, and it can be affected by various factors, including diet and lifestyle. In this context, a cross-sectional study involving 311 students examined the relationship between the MD and different aspects of the menstrual cycle. The results showed that women with low adherence to the MD experienced longer menstrual cycles compared to those with high adherence [22,122]. In another cross-sectional study analyzing a sample of 505 young women, nearly 90% of whom had at least one menstrual disorder, mostly dysmenorrhea (70.7%), interesting dietary patterns we found to be associated with menstrual pain: women with severe dysmenorrhea were found to consume highly refined products, processed meat, sugar, and foods high in saturated fats and low in fibers compared to women with moderate menstrual pain. These findings suggest that diet, particularly adherence to the MD, may play a role in reducing menstrual discomfort and promoting overall reproductive health [123].

#### 5.2.2. Menopause and Perimenopausal Age

In a cross-sectional study [124] involving 100 postmenopausal obese women, the relationship between adherence to the MD and menopausal symptoms was examined. The study revealed that women experiencing severe menopausal symptoms had lower adherence to the MD compared to those with none-to-moderate symptoms. This suggests a potential link between dietary patterns and the severity of menopausal symptoms.

Perimenopausal age, which typically includes the years leading up to menopause, also appears to be influenced by diet type, particularly regarding vasomotor symptoms (VMSs), like hot flushes and night sweats. Evidence suggests that adhering to a healthy dietary pattern, such as MD, might help in managing these symptoms. For instance, a prospective cohort study involving 6040 women aged 50–55, followed over nine years, found an inverse association between adherence to the MD and the occurrence of menopausal VMSs [125]. Similarly, a population-based cross-sectional study of 3508 Spanish perimenopausal women (average age 48.9 ± 4.0 years) found that high adherence to the MD was inversely related to overweight and obesity. Moreover, it was also linked to a better sense of well-being, which is typically challenged during the menopausal transition [126].

#### 5.2.3. Premenstrual Syndrome

PMS is a condition characterized by psychiatric and/or somatic symptoms that develop during the luteal phase of the menstrual cycle. These symptoms can significantly disrupt daily functioning and typically resolve shortly after menstruation, disturbing about 12% of women of reproductive age [127].

A review [128] highlighted that a diet low in refined carbohydrates, fats, salt, and liquor, combined with a high admission of natural nourishment rich in B vitamins, vitamin D, zinc, calcium, and omega-3 fatty acids, may help prevent the onset of PMS and reduce the severity of its symptoms. In contrast, the Western dietary pattern, along with smoking and the consumption of high-calory/fat/sugar/salt foods, has been strongly associated with an increased risk of developing and exacerbating PMS symptoms [129,130], making dietary modifications a potential strategy for managing the condition.

#### 5.2.4. Female Infertility

The World Health Organization (WHO) defines infertility as the inability to conceive after 12 months or more of regular unprotected sexual intercourse. This condition affects an estimated 8–12% of reproductive-aged couples globally [131]. However, this estimate may not fully reflect the global prevalence. Female infertility can result from a variety of causes, including physiological issues, endocrine system irregularities, and abnormalities in reproductive organs, such as EM and tubal infections. Additionally, lifestyle factors, like obesity, smoking, alcohol consumption, and unhealthy diets, especially those low in antioxidants and high in pro-inflammatory substances, also play a significant role in contributing to infertility [22].

Several studies demonstrated that higher adherence to the MD is associated with improved female fertility and a higher probability of achieving live birth [132,133,134,135]. In particular, a cohort study [136] involving 590 infertile women found that greater adherence to the MD was significantly associated with an increased number of available embryos, fertilized oocytes, and overall embryo yield. Another prospective study [137] of 244 women reported that those in the highest tertile of MD adherence had significantly higher rates of clinical pregnancy and live birth compared to those in the lowest tertile.

#### 5.2.5. Endometriosis

EM is a chronic inflammatory condition where endometrial stromal and glandular cells are found outside the uterine cavity in ectopic sites. It affects an estimated 6–10%, or even 15% according to some sources, of women of reproductive age, which translates to approximately 190 million women worldwide [138,139]. The pathology of EM involves various factors, such as hormone activity, the menstrual cycle, prostaglandin metabolism, and inflammation [140]. A key role appears to be played by Immunoglobulin G (IgG): variations in IgG glycosylation, such as the presence or absence of galactose, sialic acid [141,142], and fucosylation [143], can either stimulate or suppress immune responses. Specifically, it has been observed that a reduction in sialylation and galactosylation shifts IgG toward a pro-inflammatory state, which can exacerbate the immune response and contribute to the chronic inflammation observed in endometriosis [141,142,144,145]. The levels of agalactosylated IgG were found to be higher in both endometriosis and other inflammatory gynecological conditions compared to healthy women [142]. Since the immune system and other factors involved in the pathogenesis of endometriosis can be influenced by diet, numerous studies have investigated the effects of dietary changes, particularly the MD, due to its content of foods with antiproliferative, anti-inflammatory, antioxidant, and analgesic properties.

A review [138] highlighted the influence of the MD on EM, finding that vegetable oils and omega-3 fatty acids exhibit protective effects against the condition. Dairy products, known for their anti-inflammatory and immunomodulatory properties, may also reduce the risk of EM. The consumption of vegetables, fruits, and long-chain n-3 fatty acids appears beneficial in lowering the risk, whereas trans fats and red meat might increase it. Research suggests that dairy consumption, particularly of high-fat dairy products like cheese, may be protective against EM, while high butter intake could have the opposite effect.

An experimental study [139] focused on 35 women with EM observed that, at 3 months, and especially at 6 months, those adhering to the MD reported reduced pain related to dyspareunia, non-menstrual pelvic pain, dysuria, and dyschezia. Additionally, there was a significant positive correlation between lipid peroxidation and pain from dysuria and dyschezia.

One of the primary symptoms of EM is dysmenorrhea, characterized by a painful menstrual cycle, often affecting the lower abdomen and radiating to the inner thighs and back. This condition is common and can significantly impact a patient’s quality of life [146]. A review [147] found that a low-fat vegetarian diet was associated with increased serum SHBG levels, reduced body weight, and decreased duration and intensity of dysmenorrhea and premenstrual symptoms. Dietary influences on estrogen activity might mediate these symptom changes [147,148]. However, a cross-sectional study that investigated the relationship between adherence to the MD and primary dysmenorrhea in 311 health science students found no statistically significant association between the level of adherence to the MD and the severity of menstrual pain [122].

#### 5.2.6. Polycystic Ovary Syndrome

PCOS is diagnosed when at least two of the following criteria are met: elevated androgens, irregular or absent menstrual periods (excluding other causes), and polycystic ovaries visible on an ultrasound [149]. The initial approach to managing PCOS typically involves lifestyle changes, particularly focusing on weight loss and dietary adjustments to improve insulin sensitivity and prevent long-term health complications [22].

Adherence to the MD has been linked to a reduced risk of developing PCOS [150,151,152].

The chronic low-grade inflammation often observed in PCOS can disrupt ovarian function, leading to issues in hormone production, follicular development, and ovulation. The MD’s positive effects on ovulation may be due to its anti-inflammatory properties and components like linoleic acid, which enhances ovarian response to gonadotropins [153,154,155]. In contrast, the Western diet, which is high in simple carbohydrates and red and processed meats and low in fresh produce, whole grains, poultry, and fish, is associated with a high glycemic index and is rich in saturated and trans fats. This diet is linked to an increased risk of anovulatory infertility by adversely affecting endocrine function and ovarian reserve [153].

A randomized controlled trial [156] compared a moderately hypocaloric MD to a very low-calorie ketogenic diet over 45 days and found significant improvements in both groups, with more pronounced changes in the ketogenic diet group. Notably, 34% of women in the ketogenic diet group regained regular menstrual cycles after years of amenorrhea. Another study [157] compared the effects of an MD combined with a low-carbohydrate diet (≤100 g of carbohydrates per day) to a low-fat diet (≤40 g of fat per day) over 12 weeks. Both diets were effective, but the MD/low-carbohydrate diet led to greater improvements in BMI, LDL cholesterol, total testosterone, and luteinizing hormone levels, suggesting it as a preferred treatment for PCOS related to overweight. Recent research also supports the benefits of a modified ketogenic diet (such as the KEMEPHY diet), which combines elements of the MD with phytoextracts. This diet has shown improvements in weight, insulin sensitivity, and hormonal balance, including levels of LH, LH/FSH ratio, testosterone, SHBG, estradiol, and progesterone [158].

Table 2 shows a comparison of metabolic and hormonal parameters among women with PCOS following different dietary patterns [158,159,160].

## 6. Effect of Diet on Sexual Function in People with Metabolic Syndrome

### 6.1. Mediterranean Diet

Adhering to the MD markedly enhances sexual function in women with MS and FSD and decreases systemic vascular inflammation, evidenced by decreased CRP levels. In a randomized controlled trial [161], the FSFI was assessed by comparing 31 women who adhered to the MD with 28 women on a control diet. After two years, the MD group showed a significant reduction in CRP levels. In this analysis, the FSFI score served as the dependent variable, while BMI, waist circumference, physical activity levels, baseline FSFI score, and serum CRP concentrations were the independent variables: BMI accounted for 38% of the variance (*p* = 0.01), nutrient intake for 20% (*p* = 0.02), and CRP for 12% (*p* = 0.04), collectively explaining about 70% of the variability in FSFI changes. However, no single dietary component was linked to FSFI changes, and no specific sexual domain (desire, arousal, lubrication, orgasm, satisfaction, and pain) showed significant improvement solely due to dietary intervention, implying that overall female sexuality may benefit from lifestyle changes. The intake of several macronutrients can cause oxidative stress, leading to a proinflammatory state. Additionally, the fiber content in meals can affect cytokine levels, thus influencing inflammation. Dietary fiber, known for its anti-inflammatory properties, can help diminish oxidative stress induced by macronutrient intake. The combination of fiber and antioxidants in the MD may mitigate the temporary oxidative stress that follows food consumption [161].

In another randomized clinical trial [18] involving 65 men with MS and ED, 35 followed the MD, while 30 followed a different diet. After two years, the MD group exhibited significant improvements in erectile and endothelial functions, along with reduced systemic vascular inflammation, as indicated by lower CRP levels compared to the control group.

**Figure 2:**
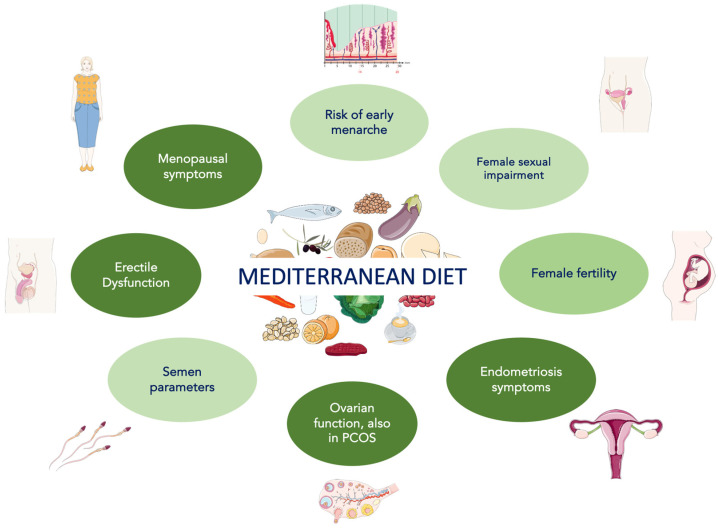
Positive effects of the Mediterranean diet on sexual dysfunction in men and women with metabolic syndrome. Light green indicates a weaker positive effect, and dark green indicates a stronger positive effect.

Figure 2 depicts the positive effects of the Mediterranean diet on sexual dysfunction in men and women with metabolic syndrome. Table 3 collects the major published studies about the influence of the Mediterranean diet on various aspects of male and female sexual function in individuals with metabolic syndrome or affected by some of its components.

### 6.2. Other Dietary Approaches and Insights on Alcohol Consumption

A randomized clinical trial assessed the impact of a low-carbohydrate diet on men with MS, specifically focusing on serum testosterone levels and erectile function [163]. The study revealed that symptoms of hypogonadism were significantly reduced in the low-carbohydrate group compared to the control group, with the percentage of affected individuals decreasing from 100% to 85.7% (*p* < 0.01). Initially, 100% of patients were hypogonadal at the beginning of the study (total testosterone serum level <300 ng/dL), but by the end of the intervention, the percentage of eugonadal men was three times higher in the low-carbohydrate group (*p* = 0.05).

A pivotal study [164] compared a low-energy diet with a high-protein, low-fat diet regarding sexual and endothelial function in obese diabetic men. It revealed significant improvements in erectile function and sexual desire and lower urinary tract symptoms (LUTSs) with both diets. The high-protein, low-fat diet also reduced systemic inflammation, indicated by lower CRP and IL-6 levels, sustaining benefits over a year.

Another study [165] examined the long-term effects of weight loss with high-protein or -carbohydrate diets on fertility and sexual function in obese men: it enrolled 118 men (body mass index, 27–40 kg/m^2^; age, 20–65 years) who were randomly assigned to an energy-restricted high-protein and low-fat (35% protein, 40% carbohydrate, 25% fat; *n* = 57) or high carbohydrate and low-fat diet (17% protein, 58% carbohydrate, 25% fat, *n* = 61) diet for 52 weeks. After one year they found similar improvements in testosterone, SHBG, and overall sexual function with both diets, though not specifically in sexual desire or erectile function.

A recent study [166] examined the effects of a very low calorie ketogenic diet on sexual function in obese patients. While no significant changes were observed in men’s sexual function, women experienced notable improvements. There were significant increases in mean total scores and the arousal and lubrication subdomains of the FSFI. Additionally, the orgasm domain showed a significant increase during the peak ketosis phase.

An open-label, parallel-group, controlled pilot trial involving obese women with PCOS and liver dysfunction found that a ketogenic diet or conventional pharmacological treatment improved menstrual cycles, reduced plasma estradiol and progesterone levels (all *p* < 0.05), and improved liver function markers compared with the control group (*p* < 0.05) [167].

In another study, moderate red wine consumption was linked to improved sexual health in women [168]. The study included 798 healthy Italian women divided into groups based on their red wine intake: moderate daily (1–2 glasses), teetotalers, and occasional drinkers. Women who consumed moderate amounts of red wine daily had significantly higher scores for overall sexual function, especially in desire and lubrication, compared to teetotalers and occasional drinkers. The study suggests that polyphenols and alcohol in red wine might improve sexual function by enhancing endothelial function. Polyphenols promote endothelium-dependent vasodilation via the NO system, increasing endothelial nitric oxide synthase (eNOS) expression, which enhances blood flow crucial for sexual arousal. Additionally, red wine polyphenols reduce endothelin-1 synthesis, a vasoconstrictor, further promoting vasodilation [168].

However, as highlighted in several reviews [169,170,171], the safety of alcohol consumption, even in moderate amounts, remains a topic of ongoing debate.

On one side, large-scale studies, such as those conducted by the Global Burden of Disease [172] and Mendelian randomization analyses [173], suggest that the only truly “safe” level of alcohol consumption is zero. These studies emphasize that even small quantities of alcohol can increase the risk of cancer, liver disease, and neurological damage, as well as having variable impacts on chronic diseases like diabetes, digestive disorders, and cardiovascular diseases (CVDs). Moreover, alcohol is linked to a heightened risk of acute conditions, including infections, injuries, traffic accidents, violence, and fetal alcohol disorders [170]. These findings support the argument that complete abstinence is the healthiest approach, advocating for a substantial reduction in alcohol intake across all population groups, regardless of their baseline consumption, age, or individual health risks.

On the other hand, some public health experts advocate for a harm-reduction strategy, particularly for middle-aged adults. This approach emphasizes the importance of avoiding binge drinking and incorporating alcohol—especially wine—within meals as part of a Mediterranean dietary pattern. Moderate wine consumption, when integrated into such a diet, has been linked to reduced all-cause mortality and a lower risk of cardiovascular disease compared to abstention or binge drinking [169,170].

Age, sex, and drinking patterns are significant modifiers of alcohol’s effects. For individuals aged 50 and above, moderate wine consumption, particularly within the context of the Mediterranean diet, continues to be associated with more favorable health outcomes, including lower all-cause mortality [169,170,171].

Conversely, younger adults (under 35) face higher risks, such as injuries, cancer, and mental health issues. For this group, the potential risks of alcohol typically outweigh any cardiovascular benefits, because the mortality attributable to excessive alcohol use represented 25% of all deaths [174].

In the absence of randomized controlled trials providing more conclusive evidence on alcohol consumption patterns, it is generally recommended that abstainers should not be encouraged to start drinking. However, for those who do consume alcohol, it is advisable to follow the Mediterranean Alcohol Drinking Pattern alongside the Mediterranean dietary pattern. Considering the addictive nature of alcohol and the practical challenges in achieving and maintaining complete abstinence (or significantly reducing consumption) among drinkers, a harm-reduction approach is recommended [169,175].

### 6.3. Adherence to the Diet Due to Personal, Cultural, or Economic Status

Adherence to dietary guidelines for managing metabolic syndrome is profoundly influenced by both cultural and economic factors, which can create significant barriers to effective dietary change. Cultural beliefs and practices play a pivotal role in shaping food preferences and dietary habits. For instance, many traditional diets, which may include high levels of carbohydrates and fats, conflict with recommended dietary patterns that advocate for the consumption of whole grains, lean proteins, and healthy fats. Studies have shown that individuals from certain cultural backgrounds may prioritize foods that have strong familial or social ties, which can hinder the acceptance of healthier alternatives [176].

Economic status further complicates dietary adherence. Individuals from lower socioeconomic backgrounds often face systemic barriers to accessing healthy food options, including higher prices for fresh produce and limited availability in their neighborhoods—commonly referred to as food deserts [177]. Additionally, economic constraints can lead to reliance on cheaper, processed foods that are typically higher in sugars and unhealthy fats, exacerbating the risk of developing metabolic syndrome [178]. The lack of nutritional education can also impede individuals’ ability to make informed dietary choices, as they may not fully understand the implications of their food choices for their health [179]

The intersection of cultural and economic factors highlights the need for culturally sensitive interventions that not only promote healthier dietary practices but also consider the socioeconomic realities faced by individuals. For instance, community-based programs that incorporate traditional foods in a healthier manner can foster greater acceptance and adherence to dietary changes [180]. Ultimately, addressing these multifaceted barriers is crucial for improving adherence to dietary recommendations, which can significantly impact the management of metabolic syndrome and its associated complications, including sexual dysfunctions [181].

### 6.4. Individuals’ Response to Dietary Interventions and Personalized Nutrition Strategies

Individuals’ responses to dietary interventions can vary widely, influenced by genetic, environmental, and psychological factors. Personalized nutrition strategies, which tailor dietary recommendations to an individual’s unique metabolic profile, preferences, and lifestyle, have emerged as a promising approach to enhancing dietary adherence and effectiveness. Such strategies may include personalized meal plans that focus on macronutrient composition, portion sizes, and specific food choices designed to optimize metabolic health [182]. Studies indicate that individuals receiving personalized dietary guidance exhibit greater engagement and are more likely to achieve meaningful weight loss and improvements in key metabolic parameters, such as reduced insulin resistance and lower triglyceride levels, compared to those following standard dietary recommendations [183].

These personalized dietary interventions have significant clinical implications for metabolic syndrome, a condition characterized by obesity, dyslipidemia, hypertension, and insulin resistance. Effective dietary management can lead to substantial reductions in body mass index (BMI) and waist circumference, improvements in glycemic control, and favorable changes in lipid profiles [184]. Moreover, these metabolic improvements can extend to sexual function, as research shows a strong link between metabolic health and sexual well-being. For instance, reduced insulin resistance and improved blood flow resulting from weight loss and better dietary habits can enhance erectile function and overall sexual satisfaction [39].

Furthermore, dietary interventions that prioritize heart-healthy fats, fiber-rich carbohydrates, and lean proteins not only support weight management but also promote hormonal balance, which is crucial for sexual health. As metabolic syndrome is often associated with erectile dysfunction and decreased libido, addressing these dietary components can lead to improved sexual function outcomes, enhancing the quality of life for individuals affected by these conditions. However, it is crucial that these interventions are coupled with ongoing support and education to ensure sustainable dietary changes and long-term health benefits.

## 7. Discussion

Sexual function is an essential aspect of overall well-being, with significant influences from lifestyle, diet, stress, and medical conditions. The WHO emphasizes that sexual health is “a state of physical, emotional, mental and social well-being in relation to sexuality” and not “merely the absence of disease, dysfunction, or infirmity” [185]. Multiple factors can deteriorate sexual function, such as partner and relationship issues and psychological problems, but medical issues surely play a significant role [44]. Modifiable risk factors include smoking, physical inactivity, obesity, and excessive alcohol and drug consumption. Hence, healthy lifestyle changes could be a useful strategy for reducing the risk of developing sexual dysfunction [186]. During the last decades, research has increasingly focused on the role of diet, particularly the MD, in modulating sexual function due to its anti-inflammatory, antioxidant, and vasodilatory properties.

The body of evidence collected in this review strongly suggests that the MD could improve sexual function both in men and women with MS. Direct evidence of this effect comes from two randomized controlled trials [18,161] that were designed specifically to assess the efficacy of the MD in people with MS and sexual dysfunctions. Nevertheless, a plethora of research studies have been conducted to investigate the efficacy of the MD on sexual dysfunction, especially in people with T2D, obesity, PCOS, and EM. Indeed, MS and sexual dysfunction are strictly interconnected, and various pathophysiological pathway characteristics of MS are demonstrated to be implied in the onset of sexual dysfunctions both in men and women.

In men, the MD has been demonstrated to improve ED and some semen parameters that could lead to the amelioration of infertility.

Among the primary organic causes of ED are metabolic–inflammatory conditions. Given that healthy dietary patterns can reduce low-grade inflammation [107], several studies have explored the link between diet and ED, with a particular focus on the MD. While the precise mechanism by which MD alleviates ED, particularly in men with MS, is not fully understood, it is hypothesized that this diet may help lower oxidative stress and subclinical inflammation, as well as enhance insulin sensitivity, which in turn could boost nitric oxide (NO) production in penile arteries [186].

One of the main culprits linking poor diet and obesity to decreased semen quality is oxidative stress, which is now recognized as a leading cause of male infertility, implicated in 30–80% of cases. Oxidative stress occurs when reactive oxygen species (ROS) overwhelm the antioxidant defenses of sperm cells, leading to damage in the cell membrane lipids, proteins, and DNA. This oxidative damage can result in reduced sperm motility, lower live sperm count, decreased sperm concentration, and higher risks of miscarriage and developmental issues in offspring. Additionally, excessive ROS production is associated with deteriorating sperm morphology. Factors contributing to this oxidative stress include the consumption of pro-inflammatory foods, low intake of antioxidant-rich foods, and diets with a high glycemic index and load [111].

In women, adherence to the MD was shown to ameliorate FSD, menstrual cycle dysfunctions (PMS and dysmenorrhea), menopausal symptoms, infertility, EM, PCOS symptoms, and ovarian function.

Female sexual health is negatively influenced by MS: neuronal and vascular injuries affecting the pelvis, caused by dyslipidemia, impaired fasting glucose (IFG), and systemic arterial hypertension can lead to female sexual dysfunction. To date, only a few studies have demonstrated a higher prevalence of sexual disorders in women with MS compared to those without [62]. The mechanisms involved are analogous to those found in men, including chronic vascular inflammation, oxidative stress, and atherosclerosis [187]. As in men, NO is important to female sexual health by increasing blood flow to the clitoris, vagina, and external sexual reproductive organs, facilitating vaginal lubrication and erectile tissue response. However, there is no strong evidence linking the lack of NO synthetase to female sexual dysfunction [188].

The MD is rich in vegetables, fruits, and cereals, which carry plenty of antioxidants and fiber that might help mitigate inflammation and oxidative stress. Those factors are crucial in EM development, but the impact of fruit and vegetable consumption on EM risk shows mixed results across studies. Dietary modifications have been shown to alleviate EM symptoms, particularly those with anti-inflammatory properties, like omega-3 fatty acids, vitamin D, and antioxidants. Surveys suggest that reducing foods that influence estrogen levels or inflammatory processes can ease symptoms, and supplements such as omega-3s, vitamins, and antioxidants may offer additional benefits. Specific supplements, like resveratrol and nutraceutical products, have been noted for their potential in reducing inflammation and pain associated with EM. Conversely, gluten and nickel might exacerbate symptoms, indicating the importance of dietary adjustments in managing EM [12,14].

The MD also affects gut microbiota composition and intestinal health [189], which may impact EM through mechanisms involving estrogen, immunity, and inflammation, making it a potential target for future treatments [190]. Research suggests that individuals with EM have altered gut microbiota profiles, including decreased diversity and increased Firmicutes/Bacteroidetes ratios. Specific bacterial taxa, like Prevotella_7 and Coprococcus_2, are more abundant in individuals with EM [191]. Furthermore, gut microbiota may affect estrogen metabolism, with certain bacteria producing enzymes that increase estrogen levels in circulation, potentially worsening EM. The interaction between gut microbiota and inflammation is crucial, as inflammation plays a central role in EM development. Research shows that gut microbiota can influence inflammatory responses and cytokine levels, contributing to the progression of the disease [190,192,193].

The MD may also benefit women with PCOS by reducing markers of inflammation and oxidative stress, improving lipid profiles and insulin sensitivity, providing cardiovascular protection, and lowering levels of visceral and subcutaneous adipose tissue, as well as waist-to-hip ratio.

Another important factor to consider is that sexual dysfunction can affect psychological well-being. Quality of life is already impaired in people with MS, and the onset of sexual dysfunction could worsen this scenario. Patients with both MS and ED experience higher levels of anxiety compared to those without MS. This increased anxiety can lead to dysfunctional behaviors, thereby perpetuating a vicious cycle [48]. Moreover, MS influences the patients’ sexual lives psychologically as well: Jagstaidt et al. found a higher percentage of sexual dysfunction and dissatisfaction in obese individuals compared to those with normal weight [44].

Given MS’s alarming actual prevalence rates and future projections not only in adults but also in children and adolescents, a feasible prevention strategy is needed to alleviate the burden of MS, along with its complications and associated conditions. MD seems to be a valid dietary approach that could improve multiple aspects of MS, including sexual function. The large-scale adoption of the MD could also have numerous positive adjunct effects on cardiovascular diseases, cancer, and chronic illness prevention: the MD has been broadly investigated for its positive effect on cardiovascular, metabolic, and mental well-being, showing significant improvements in health status, marked by a reduction in overall mortality (9%), mortality from cardiovascular diseases (9%), incidence or mortality from cancer (6%), and incidence of Parkinson’s disease and Alzheimer’s disease (13%) [194,195].

Our paper has strengths and limitations. Among limitations, the narrative fashion of the literature search and data reporting should be mentioned. Our work does not provide an exhaustive and comprehensive review of the current knowledge on the topic. Moreover, it focuses mainly on one type of nutritional pattern, the MD, and does not offer exhaustive information regarding the links between other healthy dietary approaches and an individual’s sexual health. Nevertheless, we believe that our narrative review provides interesting insight into and a rich and meaningful summary of the interrelation between MS, sexual disorders, and the impact of MD.

Future research studies should provide direct evidence of the effects of the MD on people with MS and various sexual dysfunctions. Participants should be included according to standardized inclusion criteria to limit the heterogeneity of the populations studied. Other dietary approaches, such as the ketogenic diet, or other low-carbohydrates and high-protein diets have been proven successful in improving sexual function in people with MS, but this evidence needs to be corroborated with further studies and maybe with direct comparison with the MD.

## 8. Conclusions

The available evidence suggests that adhering to the MD will enhance sexual function in MS patients, both male and female. More research is needed to corroborate this information regarding the many aspects of sexual dysfunction in both genders, as well as to compare it to other dietary treatments.

## Figures and Tables

**Table 1:** Classification of sexual dysfunctions and other relevant medical conditions according to ICD-10 and DSM-5.

Category	Dysfunction	Gender	ICD-10	DSM-5
Sexual desire disorders				
	Hypoactive sexual desire	M/F	F 52.0	Male hypoactive sexual desire disorder
	Sexual aversion and lack of sexual enjoyment	M/F	F 52.1	Deleted from DSM-5
	Excessive sexual drive	M/F	F 52.7	/
Sexual arousal disorders				
	Erectile dysfunction	M	N 48.4	Erectile disorder
	Psychogenic impotence	M	F 52.2	/
	Subjective sexual arousal dysfunction	F	F 52.2	Female sexual interest–arousal disorder
	Genital sexual arousal dysfunctions	F	F 52.2	Female sexual interest–arousal disorder
	Genital and subjective arousal dysfunction	F	F 52.2	Female sexual interest–arousal disorder
	Persistent genital arousal dysfunction	F	F 52.2	/
Orgasmic disorders				
	Premature (early) ejaculation	M	F 52.4	Premature (early) ejaculation
	Delayed ejaculation	M	F 52.3	Delayed ejaculation
	Anejaculation	M	F 52.3	/
	Orgasmic dysfunction	M/F	F 52.3	Female orgasmic disorder
	Psychogenic Anorgasmy	M/F	F 52.3	/
Sexual Pain Disorders				
	Dyspareunia	F	N 94.1	Genito-pelvic pain–penetration disorder
	Non-organic dyspareunia	F	F 52.6	Genito-pelvic pain–penetration disorder
	Vaginismus	F	N 94.2	Genito-pelvic pain–penetration disorder
	Non-organic vaginismus	F	F 52.5	Genito-pelvic pain–penetration disorder
Other Conditions				
	Endometriosis	F	N 80	/
	Polycystic ovary syndrome	F	E 28.2	/

ICD-10, International Classification of Diseases, 10th Edition; DSM-5, the Diagnostic and Statistical Manual of Mental Disorders, 5th Edition; M, male; F, female.

**Table 2:** Comparison of metabolic and hormonal parameters among women with PCOS following different dietary patterns.

Parameter	Low Adherence to Mediterranean Diet	High Adherence to Mediterranean Diet	Ketogenic Diet
CRP levels (ng/dL)	↑	↓↓	↓
Insulin sensitivity (μU/mL)	↓	↑	↑↑
Fasting glucose (mg/dL)	↑	↓	↓↓
HoMA-IR	↑	↓	↓↓
Testosterone (ng/dL)	↑	↓	↓
Ferriman–Gallwey score	↑	↓	↓
LH/FSH ratio	↑	↓	↓

CRP, C-reactive protein; HoMA-IR, Homeostatic Model Assessment Index Insulin Resistance; LH, luteinizing hormone; FSH, follicle-stimulating hormone; ↑, slight increase; ↑↑, strong increase; ↓ slight decrease; ↓↓, strong decrease.

**Table 3:** Influence of the Mediterranean diet on various aspects of male and female sexual function in individuals with metabolic syndrome or affected by several of its components.

**Males**				
**Type of Study**	**Type of Dysfunction Studied**	**Type of Influence**	**Year**	**Ref.**
RCT	Erectile dysfunction	MD improved ED in T2D patients compared to the low-fat diet	2009	[102]
Case–control study	Erectile dysfunction	High adherence to MD was inversely related to ED	2006	[107]
Prospective study	Erectile dysfunction	High adherence to MD was inversely associated with ED in 21,469 men	2020	[106]
Observational study	Erectile dysfunction	High adherence to MD showed a low prevalence of overall and severe ED	2010	[108]
RCT	Erectile dysfunction	MD in 65 men with MS improved ED compared to the control group	2006	[18]
Cross-sectional analysis	Infertility	High adherence to MD has been associated with better semen parameters	2019	[112]
**Females**				
**Type of Study**	**Type of Dysfunction Studied**	**Type of Influence**	**Year**	**Ref.**
RCT	Female Sexual Function Index	MD reduced sexual impairment in newly diagnosed T2D compared to the low-fat diet group	2009	[102]
Longitudinal cohort study	Early menarche	High adherence to MD lowered the risk of early menarche	2002	[121]
Cross-sectional study	Dysmenorrhea	Higher consumption frequency of refined cereal products, processed meat, and sugar was linked to menstrual distress	2024	[123]
Cross-sectional study	Menopausal symptoms	Low adherence to MD was linked to severe menopausal symptoms in postmenopausal obese women	2022	[124]
Prospective cohort study	Menopausal symptoms	Adherence to MD was inversely associated with menopausal vasomotor symptoms in 6040 women aged 50–55 followed over nine years	2013	[125]
Cross-sectional study	Menopausal symptoms	High adherence to MD was inversely related to symptoms of the menopausal transition in 3508 Spanish perimenopausal women	2015	[126]
Systematic review	Menopausal symptoms	Short-term adherence to a MD reduced vasomotor symptoms in peri- and postmenopausal women	2020	[162]
Systematic review	Premenstrual syndrome	A diet low in refined carbohydrates, fats, salt, and liquor and high in admission of natural nourishment rich in B vitamins, vitamin D, zinc, calcium, and omega-3 fatty acids avoided the onset and the symptoms of PMS	2024	[128]
Cohort study	Infertility	High adherence to MD was significantly associated with an increased number of available embryos, fertilized oocytes, and overall embryo yield	2019	[136]
Prospective study	Infertility	High adherence to MD was linked to higher rates of clinical pregnancy and live birth	2018	[137]
Cross-sectional	Endometriosis symptoms	Adherence to the MD was inversely related to the severity of menstrual pain in 311 healthy women	2020	[122]
Systematic review	Endometriosis incidence	MD reduced EM risk	2024	[122]
Prospective study	Endometriosis symptoms	MD reduced pain in terms of dyspareunia, non-menstrual pelvic pain, dysuria, and dyschezia in women with EM	2023	[139]
Systematic review	Endometriosis symptoms	High adherence to MD decreased pain and improved overall condition in women with EM	2022	[112]
Case–control study	Incidence of PCOS	MD reduced inflammatory state and the risk of PCOS	2022	[150]
Observational study	Incidence of PCOS	Low adherence to the MD in patients with PCOS was common in metabolically unhealthy obese ones	2021	[121]
Case–control study	Ovarian function	High adherence to the MD enhanced fertility in women aged 20–45 years who reported having difficulty getting pregnant	2011	[152]
Narrative review	Ovarian function	MD was shown to be beneficial in the regulation of ovarian function	2022	[153]
RCT	Ovarian function in patients affected by PCOS	MD determined a significant change in the anthropometric and biochemical parameters in women affected by PCOS	2023	[156]
RCT	Ovarian function in patients affected by PCOS	MD combined with a low-carbohydrate diet significantly restored the menstrual cycle in women affected by PCOS	2022	[157]
RCT	Female Sexual Function	MD was not linked to FSFI and specific sexual domains’ (desire, arousal, lubrication, orgasm, satisfaction, and pain) improvement	2007	[161]

RCT, randomized controlled trial; MD, Mediterranean diet; ED, erectile dysfunction; T2D, type 2 diabetes; EM, endometriosis; PCOS, polycystic ovary syndrome; FSFI, Female Sexual Function Index.

## Data Availability

No new data were created or analyzed in this study. Data sharing is not applicable to this article.

## Supplementary Materials

The following supporting information can be downloaded at https://www.mdpi.com/article/10.3390/nu16193397/s1, Appendix S1: PubMed search strategies.
