# Supplementary material for: Influence of Mediterranean Diet on Sexual Function in People with Metabolic Syndrome: A Narrative Review
Source: Nutrients. 2024 Oct 6;16(19):3397. doi: 10.3390/nu16193397 (PMC11479179; doi:10.3390/nu16193397)
Supplement: Supplementary file 1 [file nutrients-16-03397-s001.zip › nutrients-3215227-supplementary.pdf]

nutrients

Review

Influence of Mediterranean diet on sexual function in people with metabolic syndrome: a narrative review

Vittorio Oteri MD[1†], Francesco Galeano MD[1†], Stefania Panebianco MD[1], Tommaso Piticchio MD[1,2], Rosario Le Moli MD, PhD[1,2], Lucia Frittitta MD, PhD[1,3], Veronica Vella MD, PhD[1], Roberto Baratta MD, PhD[4], Damiano Gullo MD[4], Francesco Frasca MD, PhD[1,4*], Andrea Tumminia MD, PhD[4]

[1] Endocrinology Section, Department of Clinical and Experimental Medicine, Garibaldi-Nesima Hospital, University of Catania, Catania, 95122, CT, Italy, research@droteri.it, francesco.galeano3@gmail.com, dottoressa.panebianco@gmail.com, tommaso.piticchio@unikore.it, rosario.lemoli@unikore.it, lucia.frittitta@unict.it, veronica.vella@unict.it, frascafranco@gmail.com
[2] Department of Medicine and Surgery, University Kore of Enna, Enna, 94100, EN, Italy
[3] Diabetes and Obesity Center, Garibaldi-Nesima Hospital, University of Catania, Catania, 95122, CT, Italy
[4] Endocrine Unit, Garibaldi-Nesima Hospital, Catania, 95122, CT, Italy, rob.baratta@gmail.com, gullo.family@alice.it, andreatumminia82@gmail.com

* Correspondence: frascafranco@gmail.com
† These authors contributed equally to this work

Appendix S1. *PubMed search strategies*

Metabolic syndrome

("Metabolic Syndrome"[Mesh] OR (metabolic syndrome))

Interconnections between sexual function and metabolic syndrome

("Metabolic Syndrome"[Mesh] OR (metabolic syndrome)) AND ((sexual function) OR (sexual dysfunction) OR "Sexual Health"[Mesh] OR "Sexual Behavior"[Mesh] OR "Sexual Dysfunctions, Psychological"[Mesh] OR "Sexual Dysfunction, Physiological"[Mesh] OR erectile OR ejaculatory OR dyspareunia

OR vaginismus OR endometriosis OR PCOS OR (polycystic ovary syndrome) OR dysmenorrhea OR infertility OR menopause OR libido OR orgasm OR arousal OR satisfaction OR desire)

Impact of the Mediterranean diet on metabolic syndrome

("Diet, Mediterranean"[Mesh] OR (mediterranean diet) OR "Diet Therapy"[Mesh]) AND ("Metabolic Syndrome"[Mesh] OR (metabolic syndrome))

Impact of the Mediterranean diet on sexual function

("Diet, Mediterranean"[Mesh] OR (mediterranean diet) OR "Diet Therapy"[Mesh]) AND ((sexual function) OR (sexual dysfunction) OR "Sexual Health"[Mesh] OR "Sexual Behavior"[Mesh] OR "Sexual Dysfunctions, Psychological"[Mesh] OR "Sexual Dysfunction, Physiological"[Mesh] OR erectile OR ejaculatory OR dyspareunia OR vaginismus OR endometriosis OR PCOS OR (polycystic ovary syndrome) OR dysmenorrhea OR infertility OR menopause OR libido OR orgasm OR arousal OR satisfaction OR desire)

Effect of diet on sexual function in people with metabolic syndrome

("Diet, Mediterranean"[Mesh] OR (mediterranean diet) OR "Diet Therapy"[Mesh]) AND ("Metabolic Syndrome"[Mesh] OR (metabolic syndrome)) AND ((sexual function) OR (sexual dysfunction) OR "Sexual Health"[Mesh] OR "Sexual Behavior"[Mesh] OR "Sexual Dysfunctions, Psychological"[Mesh] OR "Sexual Dysfunction, Physiological"[Mesh] OR erectile OR ejaculatory OR dyspareunia OR vaginismus OR endometriosis OR PCOS OR (polycystic ovary syndrome) OR dysmenorrhea OR infertility OR menopause OR libido OR orgasm OR arousal OR satisfaction OR desire)
